# Results from a First-in-Human Trial of a Novel Vascular Sealant

**DOI:** 10.3389/fsurg.2015.00029

**Published:** 2015-07-01

**Authors:** Hans-Joachim Florek, Jan Brunkwall, Karl-Heinz Orend, Ian Handley, John Pribble, Ronald Dieck

**Affiliations:** ^1^Department of Vascular Surgery, Helios Kliniken Frietal, Freital, Germany; ^2^Department of Vascular and Endovascular Surgery, University Clinics, University of Cologne, Cologne, Germany; ^3^Department of Thoracic and Vascular Surgery, University of Ulm, Ulm, Germany; ^4^Tenaxis Medical, Inc., Mountain View, CA, USA; ^5^The Medicines Company, Seattle, WA, USA

**Keywords:** surgical sealant, hemostasis, vascular surgery, safety, efficacy evaluation, albumin, polyaldehyde

## Abstract

**Background:**

Suture hole bleeding from synthetic grafts presents a hemostatic challenge. The designs of many vascular sealants are not optimal (non-adherence to wet surfaces, excessive swelling, inflexible). PreveLeak™ (formerly ArterX^^®^^) is a polyaldehyde–bovine serum albumin-based sealant whose efficacy, safety, and performance were evaluated in this first-in-human study.

**Materials and methods:**

A prospective, single-arm, multicenter study was performed in patients undergoing open vascular reconstructions with prosthetic grafts. Sealant was applied to the suture line after completion of the anastomosis. The primary endpoint was the incidence of immediate sealing (without clinically significant bleeding) upon clamp release. Secondary endpoints were time to sealing, safety, and assessment of product performance.

**Results:**

Fifty-six anastomoses were performed in 32 patients. Grafts were Dacron (66% of sites), polytetrafluoroethylene (PTFE; 32%), or both Dacron and PTFE (2%). The femoral artery was the most common site of anastomosis (41% of sites). Immediate sealing after clamp release was achieved at all anastomoses (100%); 93% had no bleeding and 7% had oozing. No rebleeding occurred during 10 min of observation. The three most common adverse events were graft or bypass occlusion (*n* = 5 patients), infection (*n* = 4), and seroma (*n* = 3); none were device related. The sealant was considered easy to apply, quickly forming a soft gel, and adhering to tissue and grafts.

**Conclusion:**

PreveLeak effectively sealed anastomotic suture lines during vascular reconstruction procedures and was considered easy to use. Adverse events were consistent with those commonly observed in patients undergoing surgical procedures. These results provided the support for conducting a larger controlled clinical trial.

## Introduction

Anastomotic suture line bleeding with prosthetic grafts during vascular surgical procedures can result in blood loss and potential need for reoperation (1, 2). One hemostatic challenge encountered in patients undergoing vascular procedures is the use of synthetic graft materials [e.g., Dacron or polytetrafluoroethylene (PTFE)], which eliminates the use of electrocautery as a technique to achieve hemostasis with suture holes. Hemostasis can also be affected by use of medications commonly administered before or during surgical procedures, including heparin, warfarin, antiplatelet agents (e.g., clopidogrel), and novel oral anticoagulants (NOACs, i.e., factor Xa and direct thrombin inhibitors). The anticoagulant effects of warfarin and heparin can be reversed by administration of vitamin K and protamine sulfate, respectively; however, no treatment is available to reverse the antiplatelet effects of clopidogrel or to reverse the inhibition of factor Xa or thrombin with NOACs (3, 4). The performance of vascular surgical procedures in patients at risk for bleeding of brisk intensity has led to an increased need for robust methods for preventing suture line bleeding (5).

Four classes of surgical sealants are approved for use as adjunctive therapy with sutures or staples during vascular procedures (6). These products seal suture holes between the implanted prosthetic vascular graft or patch and the patient’s native vessel either by chemically binding to host tissue (three classes) or clot formation (one class). The four classes of sealants include cyanoacrylates, polyethylene glycols, mixtures of bovine serum albumin (BSA) and glutaraldehyde or polyaldehyde, and fibrin sealants. Although each class of sealant provides sealing, they differ in the characteristics that can limit their use; for example, the cyanoacrylate sealant (Omnex^®^ Surgical Sealant; Ethicon, Inc.) will only adhere to dry surfaces (6) and the polyethylene glycol hydrogel sealant (Coseal^®^ Surgical Sealant; Baxter Healthcare Corp.) can swell fourfold after application (7). Fibrin sealants differ from the other three classes of sealants in that they are also hemostatic agents. Tisseel™ (Baxter Healthcare Corp.) and Evicel^®^ (Ethicon, Inc.) contain human plasma-derived fibrinogen and carry a risk of transmitting infectious agents, a risk of air or gas embolism if administered with pressurized gas (8, 9), and may adhere differently to synthetic graft material compared to native vessel in the absence of blood (10).

There are two members of the BSA and glutaraldehyde/polyaldehyde class of sealants: BioGlue Surgical Adhesive (CryoLife, Inc.) and PreveLeak Surgical Sealant (formerly ArterX Surgical Sealant; The Medicines Company). Both contain BSA sourced from cattle certified as free of bovine spongiform encephalitis (but still have the potential risk of transmitting other infectious agents) and both are supplied in double-barrel syringes with mixing tips (11, 12). When BSA is exposed to glutaraldehyde in BioGlue, or to polyaldehyde in PreveLeak, the lysine residues in the BSA are crosslinked with extracellular proteins and form a mechanical seal, which is independent of the coagulation system (13, 14).

The characteristics of PreveLeak, gelling within 10–15 s, flexibility, swelling <10%, and adherence to a dry field or one where blood and blood components are present (15), suggested that it would be a useful surgical sealant for anastomotic suture line bleeding. In the first-in-human trial described herein, the safety and suture line sealing of PreveLeak were assessed after application to anastomotic sites in patients undergoing vascular surgical procedures.

## Methods

### Study design

This prospective, single-arm, multicenter, first-in-human study evaluated prophylactic sealing of suture lines at the anastomosis between native vessels and synthetic vascular grafts using PreveLeak, and was conducted at three German study sites (June–November 2007). The primary objective of this study was to evaluate the safety and performance of PreveLeak in achieving sealing at suture lines between native and synthetic vascular grafts used in the open surgical repair of large vessels, peripheral arterial bypass, or arteriovenous (AV) graft formation for hemodialysis access.

Within 7 days before the vascular surgical procedure, a screening visit was conducted to obtain pertinent medical history and evaluate patient eligibility. A complete blood count (CBC) and platelet count were obtained and current medication use was documented [also performed on the operative day (day 1) and post-procedure through discharge from the hospital]. On day 1, patients underwent open vascular reconstructions with prosthetic materials (PFTE or Dacron) according to standard practices. The use of perioperative, intraoperative, and postoperative anticoagulation was dependent on the Investigator’s discretion and not specified by the study protocol. PreveLeak was stored at 2–8°C and applied prophylactically (up to 4 mL) to the suture line after completion of the anastomosis or patch, but prior to the restoration of arterial flow. Approximately 2 min after application, arterial flow was restored by removing the clamps and observation of sealing was documented immediately following restoration of flow and 1, 3, 5, and 10 min later (timed with a calibrated stop watch). Bleeding was classified into one of four categories: no bleeding, oozing, fast flow, or spurting. Each patient was treated at one to three suture lines depending upon whether the patient was receiving a graft or patch and the number of anastomoses or suture lines. Product handling and performance were assessed using a questionnaire. Adverse events and medication use were also assessed at follow-up evaluations, 6 weeks and 3 months after the surgical procedure.

The Freiburg Central Ethics Committee gave national approval for the study and each site received supplementary approval from their hospital based or a surgical society ethics committee before proceeding (clintrials.gov ID Number: NCT02476318). The study was designed to be in compliance with guidelines on the clinical investigation of medical devices for human subjects (MEDDEV 2.7.1 and EN ISO 14155-1 and 2; 2003) and to adhere to the ethical principles set forth by the Declaration of Helsinki. Sites were to comply with applicable principles of Good Clinical Practice and all patients or their authorized representative provided written informed consent before any study-specific procedures were conducted.

### Endpoints

The primary endpoint was the proportion of patients achieving immediate suture line sealing on clamp removal, as evidenced by a lack of clinically significant bleeding. A minimum of 50% of the anastomoses were expected to be sealed immediately. The secondary endpoint was time to sealing, measured immediately after clamp removal and 1, 3, 5, and 10 min later. A minimum of 80% of the anastomoses were expected to be sealed 10 min after clamp removal. Safety was assessed by recording adverse events over the course of the study. User perception of the performance of the product was assessed by a questionnaire that asked about the amount of material available for application, the delivery system performance, the material adhesive quality and gel rate, the ease of use, and overall satisfaction with the product. There were four possible responses for each of the six questions (excellent, good, fair, or poor).

### Patients

Adult patients were eligible if they were scheduled for surgical placement of a prosthetic graft for large vessel repair, arterial reconstruction, or AV graft formation for hemodialysis access; had no child bearing potential or a negative serum or urine pregnancy test within 7 days before the procedure; and were willing and able to be contacted for ≥6 weeks of follow-up. Exclusion criteria included known hypersensitivity, contraindication, or allergic reaction to heparin, bovine, shellfish, or seafood products; history of bleeding diathesis or coagulopathy; refusal to receive blood transfusions; or concurrent enrollment in another clinical trial.

### Statistics

A sample size of 30–60 patients was considered appropriate based on the study objectives. The decision to stop patient enrollment was made after 30 patients completed the study as the observed immediate sealing rate exceeded the 50% success criterion established *a priori*. Two additional patients were enrolled after the decision to stop enrollment was made but prior to communicating the decision to Investigators.

No statistical comparisons were planned or performed. Results are presented with descriptive statistics, with analyses performed by an independent statistician (Willes Consulting Group, Encinitas, CA, USA). Adverse events are summarized by the verbatim terms that the Investigators used to describe them.

## Results

A total of 32 patients were enrolled. Most patients were male (81%) and median (range) age was 64.5 (46–86) years (Table 1). The 32 patients were treated with PreveLeak at 56 different suture sites while undergoing bypass graft (69% of patients), arteriotomy (25%), or AV graft formation for hemodialysis access (6%). The grafts were Dacron (66% of sites) or PTFE (32%), with 1 site (2%) including overlapping Dacron and PTFE grafts. The femoral artery was the most common (23) of the 53 recorded suture sites, with aortic (10), iliac (7), popliteal (4), tibial (4), carotid (2), brachial (2), and subclavian (1) arteries also utilized.

**Table 1:** **Patient demographic and surgical characteristics**.

	Patients (*N* = 32)	Suture sites (*N* = 56)^a^
**Mean (SD) age, years**	66 (10.1)	–
**Median (range) age, years**	64.5 (46, 86)	–
**Gender, *n* (%)**		
Male	26 (81)	–
Female	6 (19)	–
**Surgical procedures, *n* (%)**		
Bypass graft	22 (69)	46 (82)
Arteriotomy	8 (25)	8 (14)
AV access graft	2 (6)	2 (4)
**Graft material, *n* (%)**		
Dacron	22 (69)	37 (66)
PTFE	9 (28)	18 (32)
Dacron and PTFE^b^	1 (3)	1 (2)

*^a^One patient underwent two different procedures approximately 1 week apart and represented 4 of the 56 sites treated*.

*^b^During an arteriotomy sealing procedure in one patient, the new PTFE patch partially overlapped an existing Dacron patch. PreveLeak was applied to both patches*.

*AV, arteriovenous; PTFE, polytetrafluoroethylene; SD, standard deviation*.

### Efficacy and performance of the sealant

The primary endpoint, achieving immediate sealing without clinically significant bleeding upon the release of clamps and the restoration of blood flow, was met at 100% (*n* = 56) of the suture sites (Table 2), with 93% of the sites (*n* = 52) having “no bleeding” after clamp release and 7% of the sites (*n* = 4) having “oozing.” The effect was durable, with “no bleeding” reported for 98% of the sites (*n* = 55) at 10 min and “oozing” reported at 2% of the sites (*n* = 1). The effects of the sealant on the two different kinds of graft materials were comparable (Table 3). The time to achieving sealing without clinically significant bleeding (“no bleeding” or “oozing”) was considered immediate for all types of procedures and all graft materials (secondary endpoint). The investigators considered the ease of preparing PreveLeak, the performance of the delivery system, the gel rate, and the adhesiveness of PreveLeak “good” or “excellent” for the procedures performed in 30 of 32 patients.

**Table 2:** **PreveLeak performance by type of surgical procedure**.

	*n* (%) of sites			
	Bypass graft (*N* = 46)	Arteriotomy (*N* = 8)	AV access graft (*N* = 2)	Any procedure (*N* = 56)
**Bleeding^a^ at 0 min**				
“No bleeding”	43 (93)	7 (88)	2 (100)	52 (93)
“Oozing”	3 (7)	1 (13)	0 (0)	4 (7)
“No bleeding” or “oozing”	46 (100)	8 (100)	2 (100)	56 (100)
**Bleeding^a^ at 10 min**				
“No bleeding”	45 (98)	8 (100)	2 (100)	55 (98)
“Oozing”	1 (2)	8 (100)	2 (100)	1 (2)
“No bleeding” or “oozing”	46 (100)	8 (100)	2 (100)	56 (100)

*^a^Bleeding was categorized as no bleeding, oozing, fast flow, or spurting*.

*AV, arteriovenous*.

**Table 3:** **PreveLeak performance by type of graft material**.

	*n* (%) of sites		
	Dacron (*N* = 38^b^)	PTFE (*N* = 19^b^)	Any material (*N* = 57^b^)
**Bleeding^a^ at 0 min**			
“No bleeding”	34 (89)	19 (100)	53 (93)
“Oozing”	4 (11)	0 (0)	4 (7)
“No bleeding” or “oozing”	38 (100)	19 (100)	57 (100)
**Bleeding^a^ at 10 min**			
“No bleeding”	37 (97)	19 (100)	56 (98)
“Oozing”	1 (3)	0 (0)	1 (2)
“No bleeding” or “oozing”	38 (100)	19 (100)	57 (100)

*^a^Bleeding was categorized as no bleeding, oozing, fast flow, or spurting*.

*^b^During an arteriotomy sealing procedure in one patient, the new PTFE patch partially overlapped an existing Dacron patch. PreveLeak was applied to both patches, which are counted separately in this table*.

*PTFE, polytetrafluoroethylene*.

## Safety

A total of 30 adverse events were reported by 22 of the 32 patients up to 90 days after treatment. The events were considered by the Investigators as not related (investigator assessment of definitely or unlikely treatment-related) to treatment with PreveLeak. The most commonly reported events were graft or bypass occlusion (*n* = 5 patients), infection (*n* = 4), seroma (*n* = 3), aneurysm (*n* = 2), lymphedema (*n* = 2), and peripheral artery disease (*n* = 2). Other events were reported for only one patient each. One severe event was reported (abdominal hernia with ileus); the other events were mild (*n* = 13 events) or moderate (*n* = 16) in severity.

## Discussion

Any sealant used prophylactically for the prevention of suture hole bleeding during vascular reconstructive surgery with prosthetic grafts should be effective and fast acting to minimize blood loss and the potential need for blood transfusion and associated costs. The results from this study demonstrated that PreveLeak immediately sealed 100% of anastomotic sites in patients undergoing vascular surgical procedures, with no adverse events that could be attributed to the sealant. The sealing effect of PreveLeak was durable, as 100% of application sites remained sealed at 10 min. This sealing effect occurred independently of a patient’s coagulation cascade, was observed across a range of procedures that involved anastomoses between native vessels and Dacron or PTFE grafts, and the investigators considered PreveLeak easy to use. The most commonly observed adverse events were consistent with those often seen in patients undergoing surgical procedures or with the specific types of procedures being performed.

Time to sealing is a widely used assessment tool in clinical studies of surgical sealants, although comparisons of results between studies must be interpreted with caution due to differences in study design and/or patient populations studied. A study of Omnex showed that it immediately sealed 55% of anastomoses with synthetic grafts in femoral-popliteal bypass procedures and AV graft formation for hemodialysis access (16), while in another study, immediate sealing was achieved in 71% of patients undergoing vascular constructive surgery using PTFE grafts (17). In patients undergoing similar types of surgical procedures, Coseal achieved immediate sealing in 47% of anastomotic sites (18). Application of BioGlue resulted in immediate sealing of 61% of anastomotic sites in patients undergoing cardiac or aortic reconstructive procedures or peripheral arterial bypass (19).

PreveLeak is composed of BSA and a polyaldehyde crosslinker. Additional components of PreveLeak include chitosan chloride, which enables the sealant to adhere to surfaces that are dry or where blood and blood components are present; sodium hyaluronate, which gives the sealant high-burst strength (>500 mmHg) and an elastic modulus similar to that of healthy human aorta; and sodium carboxymethyl cellulose, which increases the viscosity of the polyaldehyde and facilitates mixing. During application, the BSA and polyaldehyde are mixed in a self-mixing syringe tip. The mixture polymerizes quickly and adheres to the application surface, which minimizes the potential for leakage through suture holes and for embolization (20). The BSA–polyaldehyde elastic, biocompatible gel forms stable crosslinks between the primary and secondary amino groups of the albumin molecules and the primary and secondary amino groups of the proteins on the surface at the application site (21) and mechanically adheres to synthetic grafts.

Reduced local toxicity is an advantage of the use of polyaldehyde over glutaraldehyde as a crosslinker because leaching of glutaraldehyde from cured BioGlue has been shown to be cytotoxic and inflammatory *in vitro* and *in vivo* (22). This inflammation may contribute to the development of pseudoaneurysm (23). In non-clinical studies, PreveLeak, which contains polyaldehyde, has been shown to be largely bioresorbed from the site of application by 12 months and was not cytotoxic in animal or clinical studies, although some cytotoxicity of extracts made from PreveLeak was observed in cell culture-based laboratory assays at concentrations higher than those likely to be encountered clinically (12, 15).

PreveLeak was designed to be compliant with the human aorta. PreveLeak’s elastic modulus of 599 kPa (data on file), a measure of its stiffness, is comparable to that reported for human aorta (455 kPa) (24). In contrast, BioGlue has a modulus value of 3,122 kPa, which is greater than that of human aorta (24). The modulus of BioGlue is also substantially greater than that of Dacron (1,100 kPa when measured circumferentially). Thus, polymerized BioGlue can contribute to complications at the anastomotic site, including stricture, because it is stiffer than both the native vessel and Dacron graft (25).

The primary limitation of the clinical study described herein was the single-arm, open-label design. The Investigator applying PreveLeak was also responsible for determining the time to sealing. Potential intra- and inter-observer variability were minimized by standardizing the assessment of time to sealing across investigative sites, and potential bias was reduced by employing independent personnel who source-verified and analyzed the data.

PreveLeak was effective in achieving rapid sealing after prophylactic application in prosthetic arterial reconstruction. It was well tolerated, with no adverse events considered device related. The sealant was easy to apply, remaining soft and flexible and firmly adherent to the application site. These results provided the support for conducting a larger controlled clinical trial, in which the performance and safety of the sealant could be more fully evaluated.

## Conflict of Interest Statement

Hans-Joachim Florek: Institution received grant for reimbursement of patient participation study costs. Jan Brunkwall: Institution received grant for reimbursement of patient participation study costs. Karl-Heinz Orend: Institution received grant for reimbursement of patient participation study costs. Ian Handley: Employee of Tenaxis Medical, Inc./The Medicines Company. Compensation included salary and stock options. John Pribble: Employee of The Medicines Company. Compensation included salary and stock options. Ronald Dieck: Former employee of Tenaxis Medical, Inc. Compensation included salary and stock options.
